# Management of irradiated post-mastectomy wound dehiscence with synthetic electrospun fiber matrix: a case report

**DOI:** 10.3389/fonc.2024.1371122

**Published:** 2024-04-18

**Authors:** Tess Montminy, Nicole E. Oppenheimer, Daniela Cocco

**Affiliations:** ^1^ Creighton University School of Medicine Phoenix, Phoenix, AZ, United States; ^2^ Department of Surgery, Valleywise Health Medical Center, Phoenix, AZ, United States

**Keywords:** irradiated wound, mastectomy, surgical dehiscence, tissue engineering, wound matrix, electrospun, synthetic

## Abstract

Breast-conserving surgery (BCS) is a well-established standard treatment option alternative to mastectomy for patients with early breast cancer that consists of a lumpectomy followed by adjuvant radiotherapy. However, irradiated tissues are at an increased risk of wound healing complications when post-treatment surgical management is required. The management of an irradiated wound dehiscence can be challenging, as it often requires a multimodal treatment approach that includes more invasive interventions when compared to a traditional surgical wound dehiscence. We present a 64 year old female patient with a remote history of right BCS with radiation therapy for early breast cancer 12 years ago, who recently required a simple mastectomy due to ipsilateral breast cancer recurrence. The post-operative course was complicated by dehiscence of the mastectomy wound. After standard wound care therapies failed, her surgical wound successfully healed after treatment with a synthetic electrospun fiber matrix application. Patients with additional comorbidities often do not qualify for invasive reconstructive options; therefore, effective local management options are warranted. This is the first reported case documenting synthetic electrospun fiber matrix efficacy and safety in healing a dehisced surgical wound within a previously irradiated fibrotic area, without the need for further invasive surgical intervention. Larger scale research, such as a prospective cohort study or randomized control trial, is needed to investigate its novel use in irradiated wounds.

## Introduction

Post-lumpectomy radiotherapy is a conventional treatment for early breast cancer following lumpectomy for patients undergoing breast-conserving surgery (BCS) (1–3). However, long-term radiation effects on tissues are well known to decrease local wound healing capabilities and increase the risk of wound dehiscence. Radiation treatment can elicit a response from the immune system that brings the wound healing process to a standstill while in the inflammatory phase. Persistent inflammation can induce a state of chronic wound healing that can evolve into radiation-induced fibrosis (4). Any additional wounds or surgeries would place that area at an increased chance of surgical site infection (SSI) and wound dehiscence. These issues alone might contribute to delayed wound healing in irradiated skin. When combined with the patient’s comorbidities, including diabetes and poor nutritional status, they unquestionably pose a considerable threat to the wound healing process.

Closing or re-approximating wound edges on irradiated skin can prove to be a significant challenge due to the underlying post-radiation tissue changes, such as thickened and stiff tissue secondary to fibrosis, ulcers, necrosis, and decreased angiogenesis (5, 6). More advanced reconstructive options can be limited due to associated patient comorbidities and extended recovery time.

Hyperbaric Oxygen Therapy (HBOT) represents a gold standard in the management of chronic/ischemic wounds due to its ability to reverse ischemia by promoting angiogenesis and improving granulation tissue formation of surgical wound beds (7). However, factors such as cost, availability, and most importantly, length of treatment (30-40 sessions) render this option less accessible when managing patients with breast cancer (8).

Among the limited minimally invasive options, the electrospun fiber wound matrix (Restrata^®^, Acera Surgical, Inc., St. Louis, MO) recently proved to be a promising synergistic tool in the management of complex wound closure. A 2022 study reported complete closure of four auricular post-Mohs wounds treated with the synthetic electrospun fiber matrix (SEFM) in patients of advanced age, with a mean healing time of 8 weeks (9). This fiber matrix does not contain exogenous growth factors, unlike biologic-based wound care, and may prove to be an ideal option for the treatment of post-oncologic wounds (10, 11).

The SEFM is an advanced wound modality with electrospun fibers composed of two synthetic polymers commonly found in absorbable sutures: polyglactin 910 and polydioxanone (12). This wound matrix is resorbable and long-lasting, as the two polymers are broken down by hydrolysis and not subject to enzymatic degradation. Several studies have examined the clinical efficacy of the treatments utilizing the SEFM in a wide range of wound etiologies, including difficult to heal wounds. The results of a 2018 study showed that 85% of 82 chronic wounds of varying wound origins, such as non-healing (diabetic, venous leg, pressure) ulcers and traumatic, vascular, necrotic, and surgical wounds, healed within 12 weeks after initial treatment with the SEFM (13). Additionally, numerous studies have demonstrated successful SEFM treatment of closing complex surgical wounds to assist in secondary healing or stimulate the formation of granulation tissue necessary for staging reconstructive procedures, such as flaps or skin grafting (13–18). However, there have been no documented clinical uses of an SEFM in irradiated tissue for enhanced wound healing.

We report a case of an irradiated post-mastectomy wound dehiscence in a patient with recurrent breast cancer previously managed with BCS and treated with an SEFM to promote complete wound healing after multiple failed wound care treatment options.

## Case description

A 64-year-old woman with a history of right breast intraductal carcinoma, managed with right breast lumpectomy with sentinel lymph node biopsy, followed by adjuvant whole breast irradiation (WBI) 12 years prior, presented to our clinic with an abnormal mammogram. Her medical history was notable for uncontrolled Type II diabetes, hypertension and hyperlipidemia.

**Figure 1:**
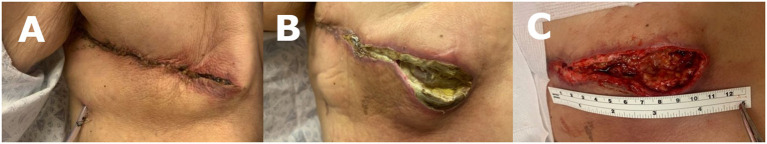
**(A)** 3 weeks post mastectomy: patient presented with chief complaint of erythema and pruritis at surgical site. **(B)** 7 weeks post mastectomy: patient returned with drainage and dehiscence of surgical incision. **(C)** 7 weeks post mastectomy: dehisced wound was debrided before NPWT treatment.

Diagnostic workup revealed a 20mm right breast lesion located at the upper outer quadrant. Biopsy of the lesion showed hormone positive invasive ductal carcinoma. At the time of consultation, blood sugar levels were noted to be elevated despite medical management (189-200 glucose, HgA1c 11.6). After the patient’s hyperglycemia was addressed by optimizing medical management, she underwent a right simple mastectomy with re-do sentinel lymph node biopsy. Intraoperatively, post-radiation fibrotic changes throughout the entire right chest wall and significant scar tissue at the former lumpectomy site were noted. A significant amount of skin flap undermining was required to achieve tension free closure. The mastectomy wound was closed with multilayered braided absorbable sutures. A surgical drain was left in place for 2 weeks. At the initial post-operative follow-up, incisional erythema was noted (**Figure 1A**), with no associated signs or symptoms of infection. Patient complained of pruritus attributed to a mild reaction to the surgical skin glue, which resolved with antihistamines. Patient was temporarily lost to follow-up and presented 7 weeks post-operative with wound dehiscence and non-purulent, serous drainage (**Figure 1B**). At that time, patient was again noted to have elevated glucose levels ranging from 200-260 with HgA1c of 10.8. The wound appeared to be poorly vascularized and was debrided down to healthy and well perfused tissues (**Figure 1C**), and a negative pressure wound therapy (NPWT) device applied.

**Figure 2:**
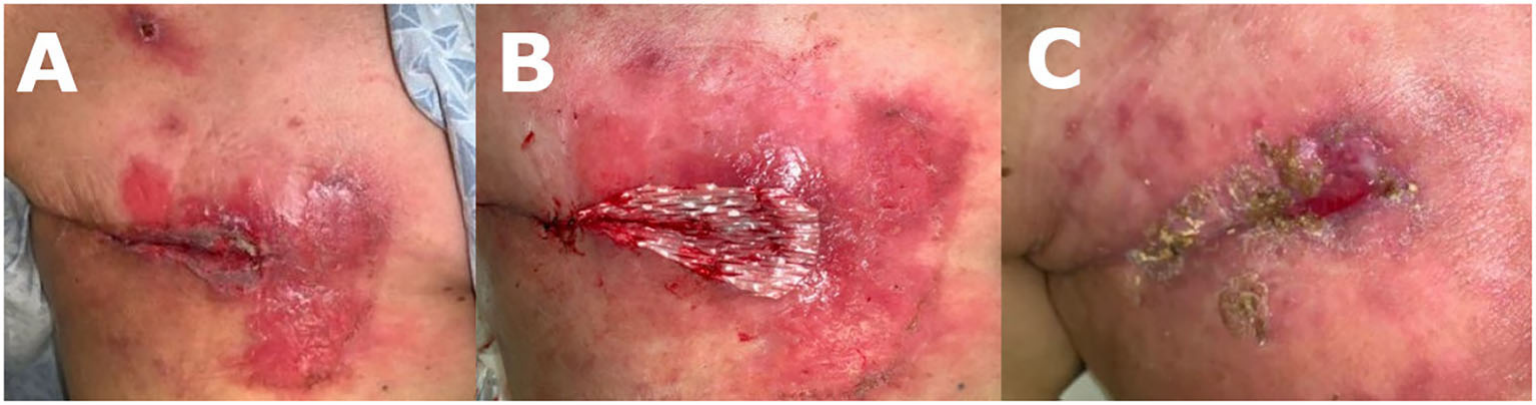
**(A)** 12 weeks post mastectomy: patient developed periwound maceration secondary to NPWT. **(B)** 12 weeks post mastectomy/SEFM week 0: first SEFM was sutured into wound bed measuring 6 x 3 cm. **(C)** 14 weeks post mastectomy/SEFM week 2: SEFM was fully integrated into the wound two weeks later, which measured at 2 x 1 cm. Second SEFM was applied to wound bed.

The decision was made to pursue alternative wound treatment after 7 weeks of NPWT, due to extensive skin maceration secondary to NPWT dressings (**Figure 2A**), the patient’s unwillingness to proceed with the treatment, and lack of availability for hyperbaric wound therapy. The wound was gently debrided, resulting in a 6 x 3 cm wound bed (**Figure 2B**). A 7.5 x 7.5 cm fenestrated SEFM was used to completely cover the wound bed while allowing minimal overlap to the wound edges. The matrix was secured in place with interrupted monofilament absorbable sutures and covered with a non-adherent dressing, also secured in place with interrupted monofilament absorbable stitches at the 4 corners of the wound bed. A bolster dressing was applied and secured to the chest with elastic retention netting.

**Figure 3:**
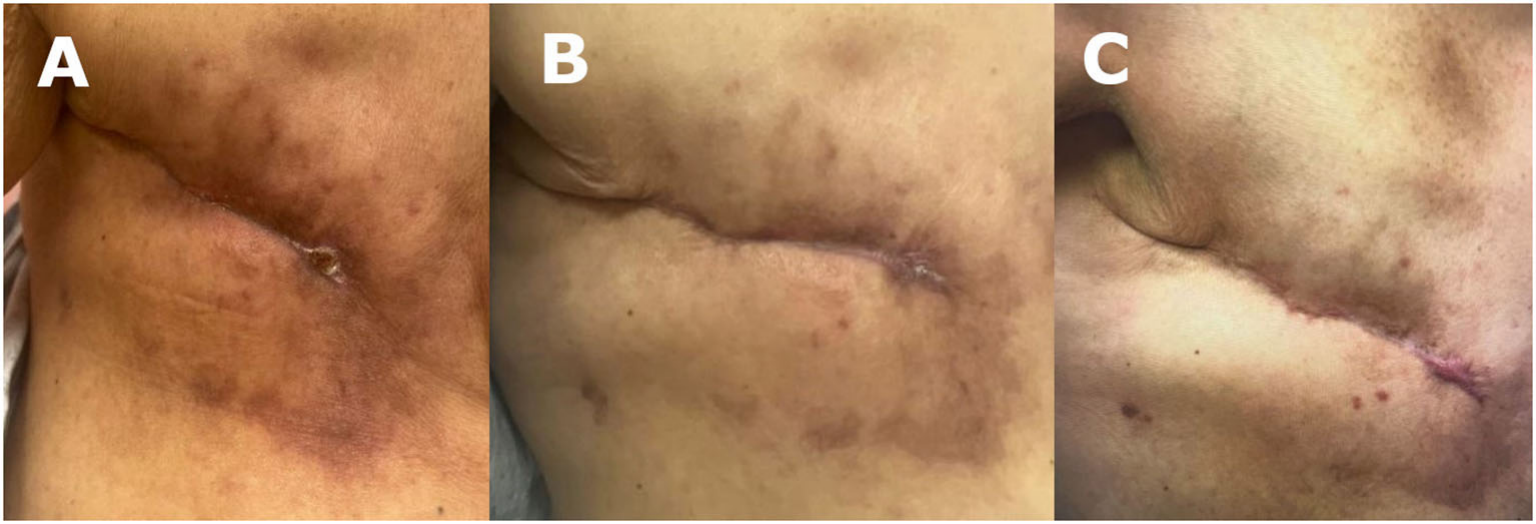
**(A)** 16 weeks post mastectomy/SEFM week 4: wound measured at 0.5 x 0.5 cm after second SEFM application. **(B)** 20 weeks post mastectomy/SEFM week 8: surgical wound was fully closed by 8 weeks after the initial SEFM application. **(C)** 36 weeks post mastectomy/SEFM week 24: wound demonstrating complete healing with no recurrence of dehiscence at 24 weeks after SEFM application.

At the 2-week follow-up, the patient exhibited improvement in skin maceration and > 50% wound contraction, with the SEFM fully incorporated into the wound bed (**Figure 2C**). The patient received a second SEFM application to the reduced wound bed (measured 2 x 1 cm), which was again completely incorporated into the wound within two weeks. Despite the persistent hyperglycemia (glucose levels ranging 250-300 and HgA1c 12), after 4 weeks, the wound was completely closed with a residual 0.5 x 0.5 cm eschar at the most medial aspect of the wound, at which point wound healing progress was sufficient, and additional SEFM applications were not warranted (**Figure 3A**). Her next two follow-up visits revealed a fully healed surgical incision at 8 weeks and 6 months, respectively, after initial SEFM treatment (**Figure 3B, C**). The 1-year surgical follow-up, 9 months following initial SEFM application, was unremarkable with no reports of dehiscence or any adverse outcomes.

## Discussion

Breast-conserving surgery followed by WBI is a well-established treatment paradigm for early-stage breast cancer. Nevertheless, ipsilateral breast cancer events after initial breast-conserving therapy pose a significant treatment challenge when a salvage mastectomy is required, due to the well-known risk for wound healing issues following radiation (19).

Skin and soft tissue changes have been reported in approximately 85-95% of patients undergoing radiation therapy (20). Radiation effects can be categorized as early (days to weeks) or delayed (several months to years) and can present with microvascular damage, tissue fibrosis, and ulcers (4). The ionization used in radiation therapy can cause structural changes to the tissues’ extracellular matrix (ECM) components by directly affecting fibroblasts, the primary contributors to collagen deposition. Dermal fibrosis induced by radiotherapy has been demonstrated to be correlated to a dysfunctional deposition of collagen, as well as an excessive differentiation of fibroblasts to myofibroblasts, which induce a cytokine mediated inflammatory response to injury (21, 22). The dysfunctional collagen production towards a predominant fibrotic tissue also affects perfusion, decreasing small blood vessel density and, consequently, tissue oxygen supply. In addition, DNA damage due to ionizing radiotherapy the production of harmful reactive oxygen species like free radicals (23), which can lead to cell dysfunction or apoptosis, leading to tissue necrosis. This can be further exacerbated by secondary damage induced by surgery, when required, leading to delayed healing or ischemic non-healing ulcers (radiation necrosis or radionecrosis).

Historically, the management of these late radiation tissue injuries (LRTI) has been unsatisfactory. Conservative treatment is usually restricted to symptom management, while definitive treatment traditionally entails a morbid surgical approach with removal of the affected area and autologous flap reconstruction (24).

To counteract the effect of late radiation-induced tissue injuries, HBOT has been suggested based upon the ability to improve angiogenesis and, therefore, increase wound perfusion. While HBOT is a widely used treatment for radiation-induced injuries (25), it comes with significant limitations. Some common barriers to obtaining HBOT include the scarcity of authorized facilities, certified practitioners, and treatment costs (26). Moreover, length of treatment and need for concomitant adjuvant therapies are the most common limiting factors for HBOT in breast cancer patients suffering with LRTI (27). In regard to this presented case, cost, availability of treatment centers, and patient’s ability tp comply with daily treatment were the main deterrents to HBOT use. As an alternative, NPWT was utilized following wound debridement. Among the various wound care options available, NPWT offers the advantage of minimizing the need for daily dressing changes, as well as aiding wound contraction while providing physical coverage. NPWT has also been proven effective in cases where comorbidities such as diabetes mellitus and previous irradiation contribute to impaired wound healing (28). However, NPWT requires skilled home healthcare assistance on a bi-weekly basis and is often complicated by skin maceration on the periwound areas, rendering this option poorly tolerated by some patients.

When compared to the standard therapies, the SEFM appears to be a promising noninvasive alternative to traditional wound management modalities with the major advantage of offering simplified wound care that is not impacted by the availability and scheduling issues of both HBOT and NPWT, allows a shorter healing time, and can be safely applied to patients with additional comorbidities.

The SEFM is a synthetic, bioresorbable, advanced wound modality that also provides wound coverage (12). In addition, the SEFM directly affects radiation-induced fibrosis by providing a scaffold for assembling new ECM and organized collagen deposition (29). Its structure is composed of fibers of varying diameters, allowing the cellular infiltration and ingrowth of critical ECM components. The SEFM has also been shown to promote neovascularization, enhancing wound healing by increasing blood flow and tissue oxygenation (9, 30). Furthermore, due to its synthetic nature, the wound matrix would be at a lower risk of stimulating an immune response, like biologic-based products.

A 2014 study examining pH and oxygenation levels of radiation patients found that irradiated skin and wounds showed higher pH values in comparison to their non-irradiated counterparts (31). As the SEFM resorbs, its byproducts reduce the pH of its immediate surroundings, creating an acidic microenvironment. The pH of healthy skin and wounds undergoing a standard wound healing process have been established to have a mildly acidic environment (12). Over time, this process creates an environment more conducive to healthy wound healing for irradiated wounds.

Additionally, when compared with the existing allogenic, xenogenic, biologic, and acellular products for irradiated wound management, the SEFM does not require complex storage temperatures or tissue tracking, while avoiding the possibility of disease transmission associated with biologic implantation, due to its synthetic nature (32).

Moreover, the SEFM has demonstrated a lower inflammatory response, faster granulation tissue formation, and more complete re-epithelialization in wounds when compared to biologic products in a preclinical study (33). Also, as the electrospun matrix degrades via hydrolysis instead of enzymatically, it has a longer persistence in the wound bed, subsequently requiring fewer clinical encounters for re-application. Faster wound closure could translate clinically to decreased delay for patients requiring post-surgical adjuvant treatment.

In the present report, since the patient could not fully comply with a strict post-operative follow-up schedule, a treatment option that would succeed without requiring frequent monitoring while providing wound coverage and lowering the risk of disease propagation was required. Once treatment with the SEFM was initiated, a significant improvement in the wound healing process was noted at her return visit two weeks later. The periwound maceration from the failed NPWT had greatly improved, as well as the patient’s discomfort. The total wound area appeared to have decreased 75% in length and 80% in depth, with remarkable re-epithelialization of the edges within two weeks after the first application of the wound matrix. Wound closure was achieved in 4 weeks, and complete re-epithelialization of the wound was completed in 8 weeks. The patient’s wound remained fully healed 6 months following the initial SEFM application, with no associated signs of hypertrophic scarring.

While LRTI are expected to be minimized with the latest, more advanced radiation therapy protocols, a significant number of patients continue to be managed with conventional WBI; as a result, the need for adjunctive wound care treatment modalities is warranted.

## Conclusion

The SEFM demonstrated its efficacy as a less invasive alternative in the management of a post-radiation wound dehiscence without any adverse effects. This case presentation is the first known reported SEFM usage in surgical irradiated wound healing. The successful clinical outcome shown in the presented report merits continued investigation into its efficacy in the treatment of dehisced wounds in irradiated tissues in other surgical fields.

## Data availability statement

The original contributions presented in the study are included in the article/supplementary material. Further inquiries can be directed to the corresponding author.

## Ethics statement

Ethical approval was not required for the studies involving humans because as a single case report containing de-identified data in the US, ethical approval from a governing research board is not required. The studies were conducted in accordance with the local legislation and institutional requirements. The participants provided their written informed consent to participate in this study. Written informed consent was obtained from the individual(s) for the publication of any potentially identifiable images or data included in this article.

## Author contributions

TM: Writing – original draft. NO: Writing – review & editing. DC: Conceptualization, Investigation, Supervision, Writing – review & editing
